# Supplementary material for: Bayesian Estimation of Muscle Mechanisms and Therapeutic Targets Using Variational Autoencoders
Source: bioRxiv. 2024 Oct 1:2024.05.08.593035. Originally published 2024 May 11. Preprint. [Version 3] doi: 10.1101/2024.05.08.593035 (PMC11100674; doi:10.1101/2024.05.08.593035)
Supplement: Supplementary file 1 [file NIHPP2024.05.08.593035v3-supplement-1.pdf]

bioRxiv preprint doi: https://doi.org/10.1101/2024.05.08.593035; this version posted October 1, 2024. The copyright holder for this preprint (which was not certified by peer review) is the author/funder, who has granted bioRxiv a license to display the preprint in perpetuity. It is made available under aCC-BY 4.0 International license.



# SUPPLEMENTARY MATERIAL

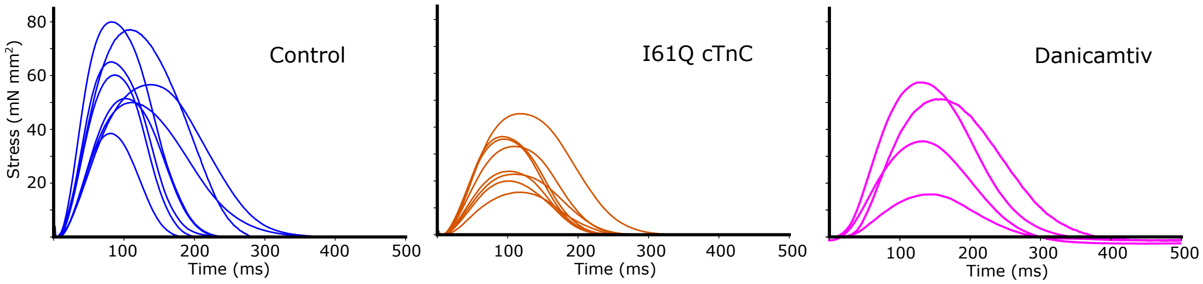


Supplementary Figure 1: Here we show twitch force in mice cardiac trabeculae from both control (blue) and mice with the I61Q cTnC variant (orange). Black dotted lines indicate the twitches from 8 different individual mice, each of which is an average of 10 twitches. The overall average is shown with 95% confidence of the mean intervals.

## Mouse Cardiac Trabeculae Isometric Twitches

Here we show the individual traces of all 8 individual muscles for both control and I61Q cTnC mouse cardiac trabeculae, as well as the 4 twitches which make up the danicamtiv dataset. Each individual trace is composed of 10 twitches which are averaged (Fig. 1). Danicamtiv data was first published in (12).

## Mean and Variance in exp data and model predictions

Because experimental data often has high variance between individual measurements, we wanted to see how the CVAE performed when shown different instances of the same type of twitch. Here we inferred the set of rates associated with the overall average twitch for a control muscle and plotted the resulting rate distribution. Then we inferred the rates for each of the 8 individual muscles separately, and then found the average rate distribution across those 8 distributions. The results show, in general, that the average of the separate predictions has a larger variance, but that the two methods produce the same means (Fig. 2).

bioRxiv preprint doi: https://doi.org/10.1101/2024.05.08.593035; this version posted October 1, 2024. The copyright holder for this preprint (which was not certified by peer review) is the author/funder, who has granted bioRxiv a license to display the preprint in perpetuity. It is made available under aCC-BY 4.0 International license.



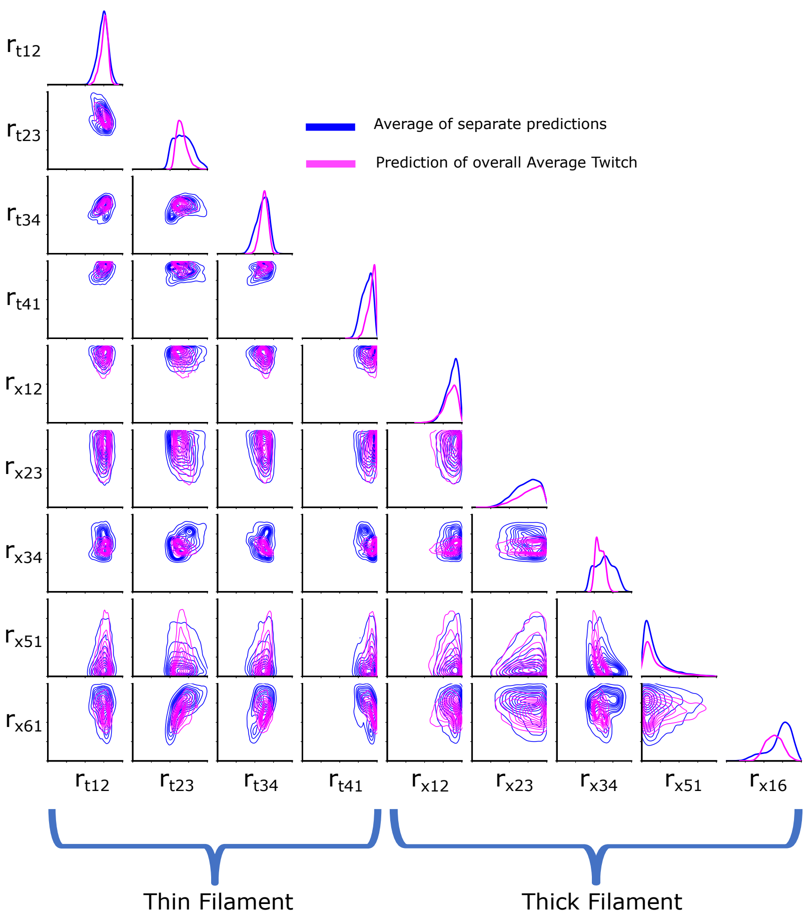


Supplementary Figure 2: Blue indicates the distribution obtained by predicting the rate distribution of each of the individual experimental twitches separately, and then averaging the results. Pink indicates the prediction obtained by first averaging the separate twitches and predicting on that target alone. All predictions were done on the control twitch set.

## Time Step Convergence

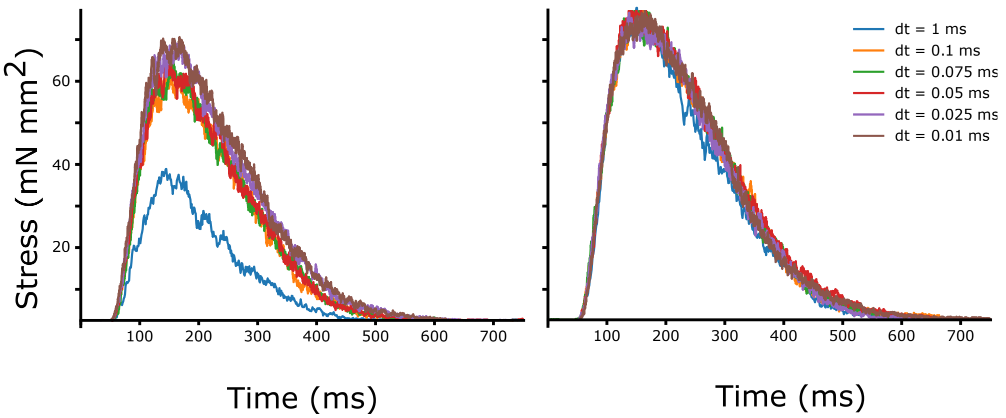


Supplementary Figure 3: To show that the probability calculations converge faster, we simulated twitches at various time steps. Left uses the old numerical scheme, and shows that even very small time steps still have not converged. Right simulations use the numerical scheme in Eq. 1, and shows approximately the same twitches regardless of time-step used.

bioRxiv preprint doi: https://doi.org/10.1101/2024.05.08.593035; this version posted October 1, 2024. The copyright holder for this preprint (which was not certified by peer review) is the author/funder, who has granted bioRxiv a license to display the preprint in perpetuity. It is made available under aCC-BY 4.0 International license.



## Heterozygous vs Homozygous

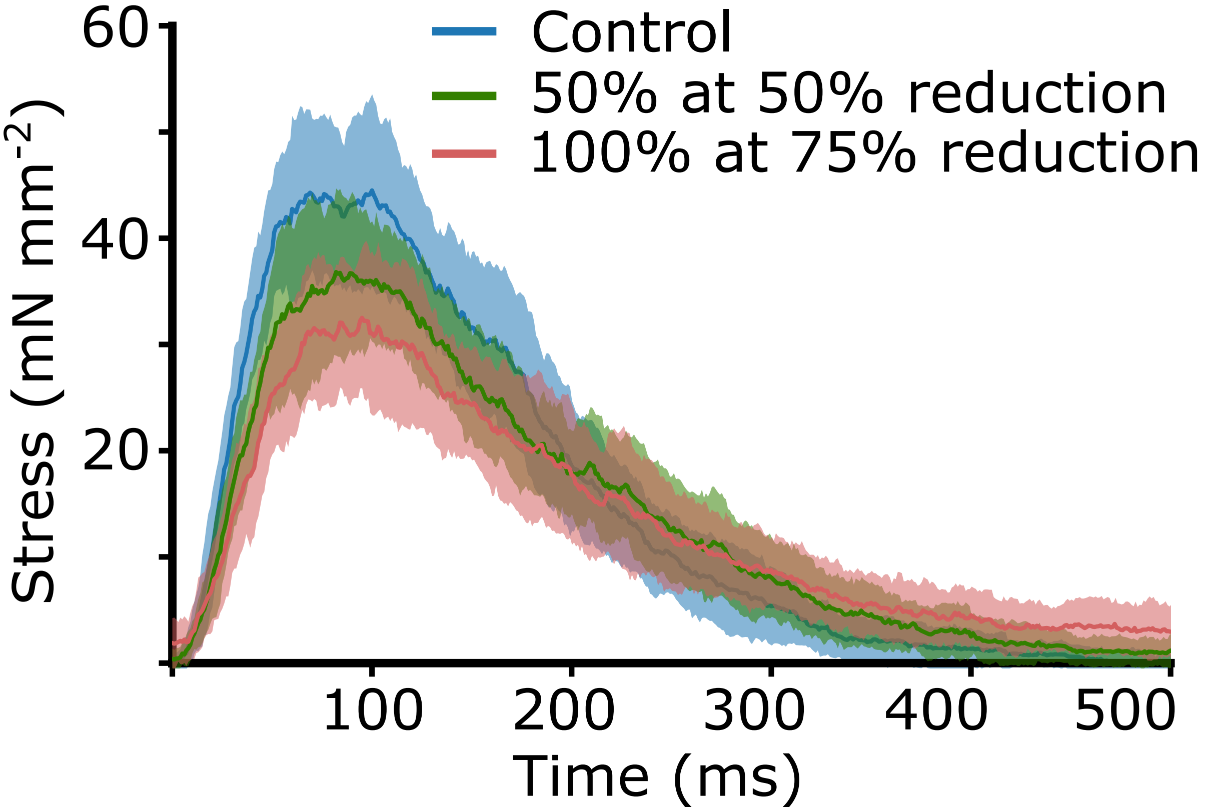


Supplementary Figure 4: Because our muscle model is spatially explicit, meaning each crossbridge and binding site are treated individually, we can modify populations of crossbridges to simulate heterozygous genetic variants. Here we show a control simulation, using the same rate constants as the Control in Fig. 7. We then performed simulations in which $r_{t,12}$ and $r_{t,14}$ had been reduced by 50% for 50% of the binding sites (teal), and another simulation in which 100% of binding sites had $r_{t,12}$ and $r_{t,14}$ reduced by 25% (brown), therefore giving the same overall average reduction of 25%. But because of the spatially explicit nature of the model, the simulations result in different twitches.

bioRxiv preprint doi: https://doi.org/10.1101/2024.05.08.593035; this version posted October 1, 2024. The copyright holder for this preprint (which was not certified by peer review) is the author/funder, who has granted bioRxiv a license to display the preprint in perpetuity. It is made available under aCC-BY 4.0 International license.



## Kolmogorov-Smirnov test

| | Control and I61Q cTnC | | | Control and Danicamtiv | | |
|---|---|---|---|---|---|---|
| | KS Statistic | KS Location | Sign | KS Statistic | KS Location | Sign |
| $r_{t,12}$ | 0.51 | -0.25 | -1 | 0.12 | -0.79 | -1 |
| $r_{t,23}$ | 0.29 | -0.011 | 1 | 0.06 | -0.55 | -1 |
| $r_{t,34}$ | 0.43 | 0.60 | 1 | 0.07 | -0.08 | -1 |
| $r_{t,41}$ | 0.49 | 0.19 | -1 | 0.13 | 0.99 | -1 |
| $r_{x,12}$ | 0.37 | 0.76 | -1 | 0.02 | -0.78 | 1 |
| $r_{x,23}$ | 0.31 | 1.26 | -1 | 0.06 | -0.34 | -1 |
| $r_{x,34}$ | 0.36 | -0.24 | 1 | 0.31 | -0.65 | -1 |
| $r_{x,51}$ | 0.21 | -0.33 | 1 | 0.08 | 0.98 | 1 |
| $r_{x,16}$ | 0.84 | -0.75 | 1 | 0.48 | -0.15 | -1 |

Supplementary Table 1. The Kolmogorov-Smirnov test is used to show how similar or dissimilar two probability distributions are. The KS statistic shows the maximum difference (supremum) between two cumulative distribution functions (CDFs), and the KS Location indicates the rate factor at which the supremum occurs. The sign indicates which CDF was larger, with negative indicating that the value of the CDF for the control type was smaller, consistent with the bulk of the probability distribution's mass occurring at larger rate factors, and positive indicating the opposite. The $p$-value in each pair-wise comparison was less than $10^{-5}$.
