# Supplementary material for: Identifying mechanisms and therapeutic targets in muscle using Bayesian parameter estimation with conditional variational autoencoders
Source: bioRxiv. 2024 May 11:2024.05.08.593035. Preprint. [Version 1] doi: 10.1101/2024.05.08.593035 (PMC11100674; doi:10.1101/2024.05.08.593035)
Supplement: Supplementary file 1 [file NIHPP2024.05.08.593035v1-supplement-1.pdf]

bioRxiv preprint doi: https://doi.org/10.1101/2024.05.08.593035; this version posted May 12, 2024. The copyright holder for this preprint (which was not certified by peer review) is the author/funder, who has granted bioRxiv a license to display the preprint in perpetuity. It is made available under aCC-BY 4.0 International license.

Supplement

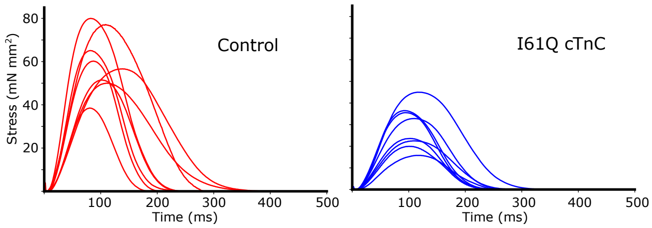

Fig. 10. Here we show twitch force in mice cardiac trabeculae from both control (blue) and mice with the I61Q cTnC variant (orange). Black dotted lines indicate the twitches from 8 different individual mice, each of which is an average of 10 twitches. The overall average is shown with 95% confidence of the mean intervals.

Mouse Cardiac Trabeculae Isometric Twitches. Here we show the individual traces of all 8 individual muscles for both control and I61Q cTnC mouse cardiac trabeculae. Each individual trace is composed of 10 twitches which are averaged (Fig. 10).

Mean and Variance in exp data and model predictions. Because experimental data often has high variance between individual measurements, we wanted to see how the CVAE performed when shown different instances of the same type of twitch. Here we inferred the set of rates associated with the overall average twitch for a control muscle and plotted the resulting rate distribution. Then we inferred the rates for each of the 8 individual muscles separately, and then found the average rate distribution across those 8 distributions. The results show, in general, that the average of the separate predictions has a larger variance, but that the two methods produce the same means (Fig. 11).

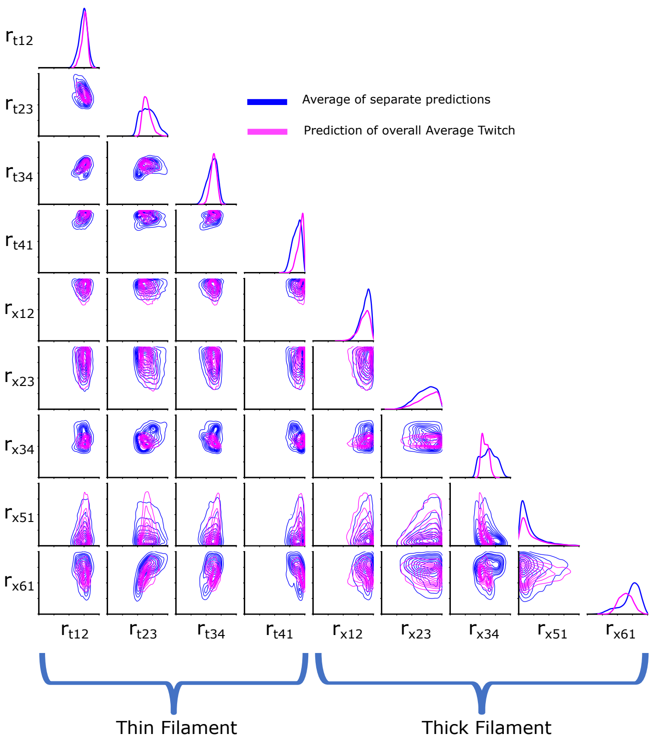

Fig. 11. Blue indicates the distribution obtained by predicting the rate distribution of each of the individual experimental twitches separately, and then averaging the results. Pink indicates the prediction obtained by first averaging the separate twitches and predicting on that target alone. All predictions were done on the control twitch set.
